# The Evolution and Genetics of Virus Host Shifts

**DOI:** 10.1371/journal.ppat.1004395

**Published:** 2014-11-06

**Authors:** Ben Longdon, Michael A. Brockhurst, Colin A. Russell, John J. Welch, Francis M. Jiggins

**Affiliations:** 1 Department of Genetics, University of Cambridge, Cambridge, United Kingdom; 2 Department of Biology, University of York, York, United Kingdom; 3 Department of Veterinary Medicine, University of Cambridge, Cambridge, United Kingdom; University of Alberta, Canada

## Abstract

Emerging viral diseases are often the product of a host shift, where a pathogen jumps from its original host into a novel species. Phylogenetic studies show that host shifts are a frequent event in the evolution of most pathogens, but why pathogens successfully jump between some host species but not others is only just becoming clear. The susceptibility of potential new hosts can vary enormously, with close relatives of the natural host typically being the most susceptible. Often, pathogens must adapt to successfully infect a novel host, for example by evolving to use different cell surface receptors, to escape the immune response, or to ensure they are transmitted by the new host. In viruses there are often limited molecular solutions to achieve this, and the same sequence changes are often seen each time a virus infects a particular host. These changes may come at a cost to other aspects of the pathogen's fitness, and this may sometimes prevent host shifts from occurring. Here we examine how these evolutionary factors affect patterns of host shifts and disease emergence.

## Introduction

Emerging infectious diseases affecting humans, wildlife, and agriculture are often the result of a pathogen jumping from its original host into a novel host species. This can take the form of spillover events that result in dead end infections or short stuttering transmission chains, or a host shift with successful infection and sustained transmission in the new host (Box 1). Host shifts have resulted in multiple human pandemics, such as HIV from chimps [1] and the H1N1 “Spanish flu” from birds [2], which have both killed tens of millions of people. Other important human pathogens have originated from other host species, including *Plasmodium falciparum* [3], SARS coronavirus [4], Hendra and Nipah viruses [5], and the measles virus [6]. Past host shifts can be detected when the phylogenies of hosts and their pathogens are different (phylogenetic incongruence—Box 1). This is very common, with a survey of the published literature finding 93% of studies comparing host and pathogen phylogenies showed evidence of host shifts [7], and there are relatively few cases where the pathogen phylogeny mirrors that of its host completely [8].

**Box 1. Glossary**

**Host shift.** We define a host shift as a parasite shifting to infect a new species of host.

**Spillover infection.** An infection that results in a dead-end infection with no onward transmission or a stuttering chain of limited transmission in the new host.

**Phylogenetic incongruence.** If the topologies of host and parasite phylogenies are not the same, it suggests that the parasites have switched between host species during their evolution.

**Clade.** A group of related species with the same common ancestor (they are monophyletic).

Here we examine the evolutionary factors that affect a pathogen's ability to infect a novel host and then discuss how the ability of a pathogen to adapt to be transmitted efficiently by a novel host can allow its long-term persistence. Following a host shift, selection will favour mutations that allow a pathogen to (a) enter a host cell with greater efficiency and (b) “fine tune” or optimise their fitness in the new host, for example by better utilising cellular machinery, enhancing immune avoidance, optimising virulence, and maximising transmission potential. Our focus is on viruses, owing to a wealth of recent studies, and because RNA viruses are the most likely group of pathogens to jump between hosts, possibly because of their ability to rapidly adapt to new hosts [9]–[12]. While we focus on genetics in this review, behaviour and ecological processes are clearly a hugely important factor in determining whether a novel host is exposed to a novel pathogen, and whether onward transmission occurs [10], [13]–[15].

## Variation in Susceptibility across the Host Phylogeny

**Figure 1:**
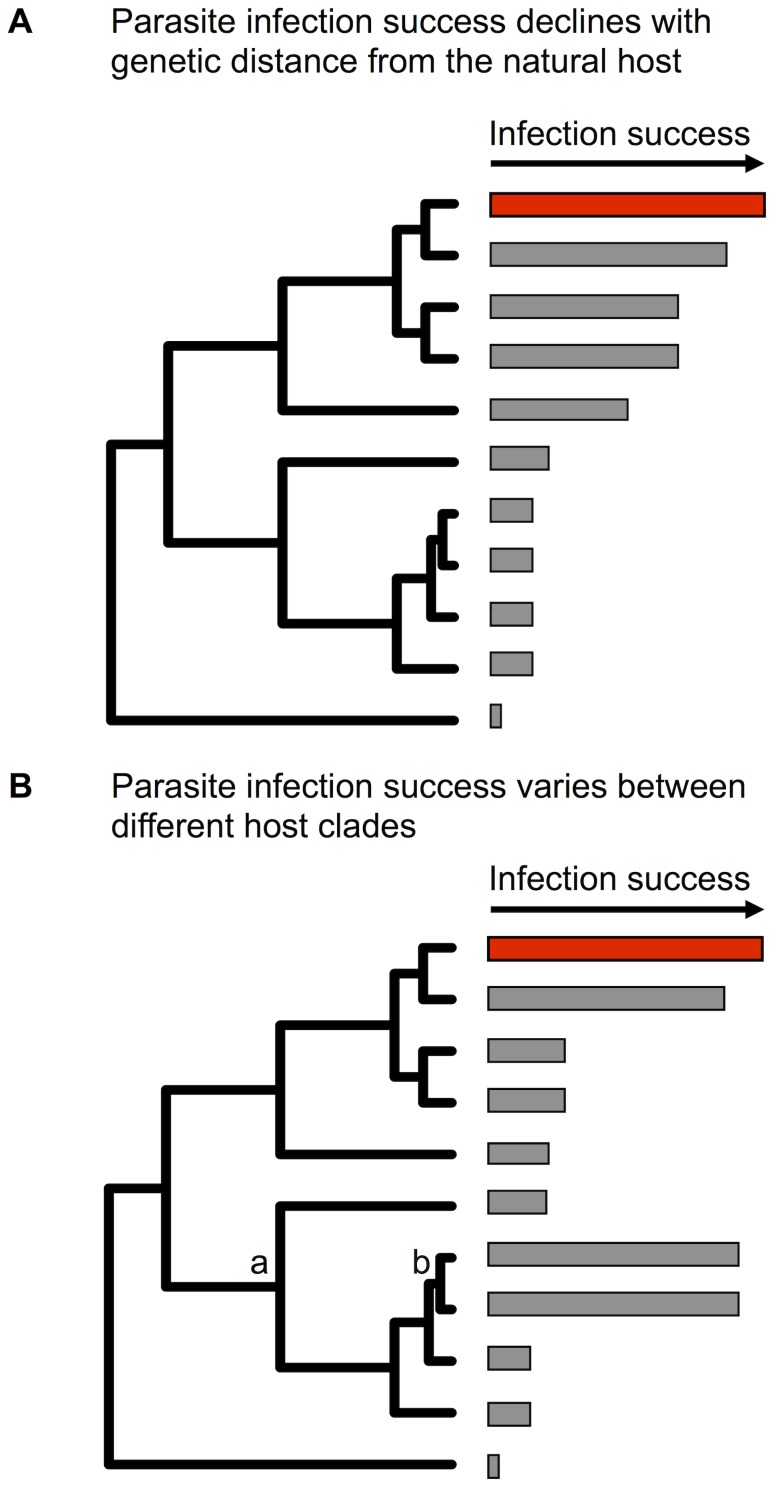
Two ways in which host relatedness may effect a pathogen's ability to host shift. The bars at the tips of the trees show a measure of pathogen infection success, with the bar in red representing the pathogen's natural host species. (A) The pathogen is less successful in host clades more distantly related to its natural host. (B) “Patches” of highly susceptible—or highly resistant—clades of hosts, may be scattered across the host phylogeny independently from their distance from the natural host. All of the species in the clade labelled “a” are equally distantly related from the pathogen's natural host. However, the species in the clade marked “b” are highly susceptible, despite being distantly related to the natural host.

The susceptibility of potential hosts varies enormously, and an important predictor of susceptibility is how closely related a novel host is to a pathogen's natural host (Figure 1A). This “phylogenetic distance effect” has been repeatedly found using experimental cross infections in all major pathogen groups, including studies of fruit flies and viruses [16], plants and fungi [17], [18], beetles and *Spiroplasma* bacteria [19], insects and *Wolbachia* [20], and fruit flies and nematodes [21]. This is presumably because close relatives of the natural host offer a similar environment to that which the pathogen is adapted to. This is likely to be especially important for pathogens because of the myriad of molecular interactions pathogens have with their hosts to infect cells, utilise resources, and avoid or suppress the host immune response.

Reconstructions of host shifts in nature have confirmed that pathogens are more likely to shift between closely related species. By reconstructing the phylogeny of rabies viruses isolated from various species of bat in North America, it has been possible to look at the patterns of cross species transmission in the wild. The rate of cross species transmission was greatest for closely related species [22] whether looking at spillover events (recent infections that might not persist long-term) or host shifts that successfully became established [13]. Similarly, viruses and other parasites of mammals are most likely to be shared by more closely related hosts [10], [23], [24], [25], [26]. Additionally, the phylogenies of hantaviruses and their rodent and insectivore hosts show evidence for host switching, with data suggestive of preferential shifts between closely related species [27]. However, within these examples there are cases of pathogens transferring successfully over great phylogenetic distances [28], [29].

Closely related species may also have similar levels of susceptibility, regardless of their distance from the pathogen's natural host (Figure 1B) [16], which we call the “phylogenetic clade effect.” Such effects could be due to certain host clades having lost or gained immune or cellular components that affect susceptibility to a given pathogen [30]. This may mean that the host phylogeny is a patchwork of clades with varying levels of susceptibility, with clades of susceptible hosts scattered across the tree, sometimes in taxa distantly related to the pathogen's natural host. This has been demonstrated in experimental cross infections of fruit flies and sigma viruses [16], where after accounting for distance from the viruses' natural hosts, the effect of the host phylogeny explained almost all of the remaining variation in viral load. If this pattern is common, it may explain cases where viruses and other pathogens recurrently shift between distantly related taxa, such as transmission of influenza viruses among birds, pigs, and humans [2], or human to bovid transmission of *Staphylococcus aureus* [31], although host ecology likely also plays a role in these instances.

The strength of these effects of the host phylogeny varies between pathogen groups, with RNA viruses and pathogens that already have a broad host range being particularly prone to jumping between distantly related species [10], [12], [32].

At the molecular level, the availability of suitable cell surface receptors to allow viruses to enter cells may be a cause of phylogenetic effects on host shifts. For example, the ability of an avian influenza virus to infect a host is initially, at least partly, determined by the presence and within-host distribution of α2,3-linked host sialic acid (SA) receptors [33].

## The Importance of Viral Entry

Some pathogens may already be pre-adapted to a novel host, but specific mutations are often required to enhance a pathogen's fitness in the new host if it is to establish successfully. A diversity of traits may change to adapt the pathogen to its new host, such as the efficiency of replication, and avoidance or suppression of host immunity, but the binding of host cell receptors is commonly especially important.

In bacteriophage (viruses of bacteria), mutations that enhance a virus' ability to bind to host cells are important in determining a virus' ability to infect a host. One mechanism is spontaneous mutations, which typically change host range by altering amino acid sequences in host-binding proteins, including tail fibres and capsid proteins [34], [35]. Similarly, experimentally evolved phage selected to infect previously resistant bacterial genotypes or different bacterial lineages also acquire mutations in genes encoding host-binding proteins [36]–[38].

In vertebrates, the importance of receptors is supported by an analysis of 64 human viruses, which found that those with the broadest host range used receptors whose amino acid sequences are the most conserved [39]. Furthermore, changes in the ability to bind host cells can also be essential for host shifts by viruses of vertebrates. This is the case for the influenza virus, which binds to sialic acid receptors, of which there are two basic types (SAα-2,3- and SAα-2,6-Gal-terminated saccharides) [33]. Avian influenza viruses bind SAα-2,3 receptors in the respiratory and gastrointestinal tracts of birds. Conversely, human seasonal influenza viruses predominantly bind to SAα-2,6 receptors in the upper respiratory tract of humans. However, humans possess SAα-2,3-sialic acid receptors in the lower respiratory tract. Though it is relatively difficult for influenza viruses to get into and out of the lower respiratory tract, avian influenza viruses do occasionally infect humans. Such infections typically result in severe disease with little or no secondary transmission. Successful adaptation to humans requires mutations to the SA receptor binding site in the hemagglutinin gene that allow the virus to utilise SAα-2,6 receptors and thus increase the potential for efficient transmission between humans [33]. A related example of the importance of changes in receptor binding is the switch of parvoviruses from cats to dogs, which was due to two mutations in the viral capsid gene that allow it to bind the canine transferrin receptor [40].

## Mutating to Adapt to Novel Hosts

If pathogens need to adapt for a host shift to be successful, then the risk of a host shift will depend on the likelihood that the necessary set of mutations can accumulate in the newly infected host (Table 1). If specific new mutations are essential for survival in a novel host, high mutation rates may be especially advantageous [41]. Accordingly, the high mutation rate of RNA viruses may explain why they host shift more frequently than other pathogens [12]. However, most mutations will be deleterious or lethal [42], [43], so the chances of a host shift will be maximised at an intermediate mutation rate [41], [44] (although it has been shown in a plant virus that the fraction of mutations that are deleterious can be reduced in a novel host [45]). How close the mutation rate of different pathogen groups is to this optimum for host shifting is unclear, so it is uncertain whether high mutation rates can explain why RNA viruses frequently jump between species.

**Table 1:** Factors that evolutionary theory predicts will affect the likelihood that the correct set of mutations will arise to adapt a pathogen to a new host.

Trait	Factors favouring a host shift
Number of mutations required	The fewer mutations required to adapt to a new host, the more likely it is that they will all occur
Epistasis and mutation order	If mutations have to occur in particular combinations to confer high fitness, then the chances of adaptation may be reduced
Mutational target size	If many different sites in the genome can be mutated to adapt to a new host, then the correct mutations are more likely to occur
Trade-offs	If mutations reduce other components of a pathogen's fitness, such as replication in alternative hosts, they may be less likely to spread in the pathogen population.
Mutation rate	High per nucleotide mutation rates increase the chance of specific mutations occurring, but can also slow rates of adaptation as many mutations are deleterious
Recombination rate	Genetic exchange, such as the exchange of plasmids, homologous recombination, and the reassortment of viral genomes, can allow the acquisition of adaptations to new hosts
Effective population size (*N_e_*)	Natural selection is more effective when the effective population size is large, and large *N_e_* populations generally have more standing genetic variation, which can accelerate adaptation
Generation time	Short generation times can increase rates of adaptation

Such theoretical predictions as listed above have been shown to be important for adaptation per se [87], and these population genetic parameters will also be important in determining the ability of a pathogen to adapt to a novel host.

The probability of a host shift will also depend on the number of mutations required to adapt to novel hosts. At one extreme, a single mutation allowed Venezuelan equine encephalitis virus to replicate efficiently in horses when it switched from rodents in the early 1990s [46]. Similarly, single mutations can underlie the expansion of the host range of RNA phages [47]. In contrast, five amino acid changes are predicted to be required for some Avian A/H5N1 influenza viruses to acquire the ability to be transmitted between ferrets [48]. If multiple mutations are required to successfully host shift, their availability can impose a constraint on host range evolution. This is illustrated by experiments on a DNA phage of *Pseudomonas fluorescens* [36]. The phage rapidly evolved to infect certain host genotypes but never adapted to others. The successful shifts were associated with one to three mutations in genes affecting host binding, whilst more mutations were required to infect the other hosts (these could be acquired through a process of coevolution; see below).

If multiple mutations are required, then the number of ways that the mutations might be fixed is important; are mutations adaptive in all genetic backgrounds, or does fitness epistasis require that they fix simultaneously or in a particular order? There have been several outbreaks of Chikungunya virus linked to the virus shifting from being predominantly vectored by the mosquito *Aedes aegypti* to *Aedes albopictus*. A single amino acid change adapts the virus to the new vector [49], but despite *A. albopictus* being common in Asia, this mutation did not occur for 60 years, and when it did, it was in a Chikungunya virus lineage of African origin. Here, the host shift mutation had no effect in the genetic background of the Asian strains, due to an epistatic interaction with a single amino acid difference elsewhere in the genome [49].

The likelihood that the mutations required to adapt to a novel host occur will also depend on the size of the mutational target in the pathogen—the number of different potential changes in the pathogen genome that adapt it to the new host. In some viruses it is common to see the same parallel mutations occurring each time a virus adapts to a particular host species [50], [51], suggesting there may be limited molecular solutions to infecting a new host species (i.e., the more parallel changes observed, the smaller the mutational target). This may act as a significant constraint on host shifts by reducing the supply of adaptive mutations.

The size of the mutational target has been most studied in phage, where host adaptation can be highly parallel, such that the same mutations become fixed in independent replicate populations [51], [52]. The importance of these mutations is illustrated by experiments that “rewind the evolutionary tape” by adapting host-shifted phages back onto the ancestral host and observe reversion mutations restoring the sequence back to the ancestral state [38]. When 40 host range mutants of phi6 phage were isolated, it was found that there were 17 unique mutations underlying this shift [35]. Furthermore, it was estimated that 56 different mutations in the phage genome have the potential to adapt it to the novel host [35]. Therefore, despite some parallelism, in this case the size of the mutational target is significant, but the possible number of combinations is large, so genetic constraints on this host shift are relatively weak.

**Figure 2:**
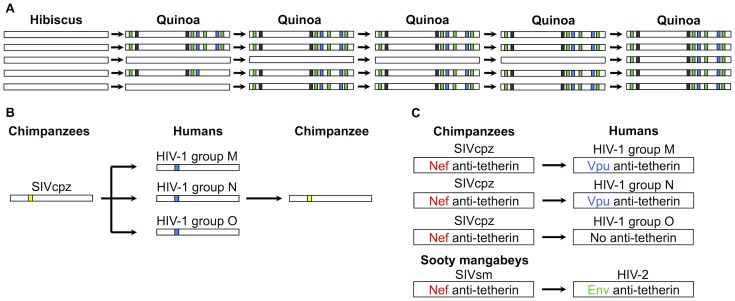
Examples of parallel adaptations following host shifts. (A) Parallel genetic changes in five replicate lines of Hibiscus chlorotic ring spot virus. The white boxes represent the viral genome, and the coloured blocks represent mutations. The virus naturally infects *Hibiscus* plants, but following five passages in an alternate host, (*Chenopodium quinoa*) the same eight mutations repeatedly occur [57]. (B) Parallel genetic changes in codon 30 of the gag gene (Met to Arg) following three independent transfers of SIVcpz into humans [59]. When a chimp was subsequently infected with HIV-1, the residue reverted back to Met. The coloured blocks represent either a Met (yellow) or Arg (blue) at codon position 30 in the HIV gag gene. (C) Parallel changes in protein function following independent transfers of SIVs from chimpanzees (HIV-1) and sooty mangabeys (HIV-2) into humans. SIV Nef protein does not antagonise tetherin in humans, and so other HIV proteins have evolved the ability to antagonise tetherin [64]. The exception to this is HIV-1 group O viruses, which do not appear to have evolved anti-tetherin activity. In HIV-1 group N viruses the evolution of anti-tetherin activity in Vpu may have come at a cost, as Vpu no longer degrades CD4 receptors to aid the release of viral particles [61]. The coloured gene names in the schematic represent the gene that provides the anti-tetherin function in that host and viral lineage.

Parallel evolution at the molecular level is also common in experiments using viruses of eukaryotes (Figure 2A). When vesicular stomatitis virus is evolved in human or dog cells, parallel mutations tend to occur within the same cell type [50]. Similarly in plants, experimental evolution of Tobacco etch potyvirus on four host species found that parallel mutations only occurred when viruses were passaged in the same host species [53]. A number of other studies in plants have found parallel mutations occurring, often after only a few passages on the novel host [54]–[57]. Experimental studies finding parallel mutations often enforce transmission and so bypass the critical barrier of successful transmission in the new host. Therefore, looking at the mutations that occur in chains of natural transmission is an important future direction, as, for example, the size of the mutational target may be different for cell entry compared to transmission [58].

Host shifts into humans have also involved parallel mutations (Figure 2B). In HIV-1, codon 30 of the gag gene has independently undergone the same change in all three human lineages of the virus that transferred from chimps [1]. This change increases viral replication rate in human cells [59], and when a chimp was infected with HIV-1 it reverted to the residue seen in SIVcpz [59]. Similarly, five parallel mutations have been observed in two independent epidemics of SARs coronavirus following the shift from palm civets to humans [60].

Parallel phenotypic changes may sometimes have different solutions at the molecular level. This has occurred in different HIV lineages that shifted into humans from other primates (Figure 2C). HIV-1 group M, which is responsible for the global pandemic, arose following a host shift by the chimpanzee virus SIVcpz [1]. In both human and chimp cells a restriction factor called tetherin can prevent the release of viral particles from infected cells [61]. In chimps, the SIVcpz protein Nef has anti-tetherin activity, but this is ineffective in humans due to a deletion in the cytoplasmic tail of tetherin that is targeted by Nef. Instead, in HIV-1 group M, the Vpu protein has evolved to perform the same function [61]. This was paralleled in HIV-2, which originates from sooty mangabeys (Figure 2C). The sooty mangabey virus also uses Nef as a tetherin antagonist and again this is ineffective in humans, but in this case the function has been acquired by the Env protein (HIV-2 lacks the Vpu protein) [61].

In cases where mutational targets are small and multiple mutations are required, several factors may help overcome mutational constraint and allow host shifts to occur. For example, the ability of avian influenza to establish infections in the eyes and lower respiratory tract of humans [33] may give time and sufficient population size for the mutations that facilitate efficient human-to-human transmission to arise. In laboratory studies of interactions between DNA phage and bacteria, it has been found that reciprocal coevolution allows phage to build up broad host ranges through the stepwise accumulation of multiple mutations in genes associated with host binding [36]. Additionally pathogens may circulate in host populations at low levels before becoming a detectable outbreak, and this may provide time for the pathogen to evolve adaptations to optimise its fitness in the novel host [62].

## Trade-offs

Adapting to a new host may have deleterious effects on other pathogen traits, and such trade-offs may reduce the chances of a host shift occurring. A possible example of this is seen in the shift of HIV-1 from chimpanzees to humans. HIV-1 group M and HIV-1 group N have independently shifted from chimpanzees to humans [1], and in both cases the Vpu protein has evolved to antagonise the restriction factor tetherin (see above). Vpu also binds and degrades CD4 receptors in SIV to aid the release of viral particles [63], but this function has been lost in group N viruses, possibly as a pleiotropic consequence of the protein gaining anti-tetherin activity [64]. It has been speculated that this may explain why HIV group N has remained a rare pathogen in Africa, while HIV group M—where the ability to degrade CD4 was retained—has become a pandemic [64].

A common trade-off of adapting to novel hosts is that performance on the original host is reduced. For example, host range mutations in the P3 protein of the RNA phage phi6 generally reduce growth on the ancestral host, although rare, cost-free mutations do exist [35], [65]. Observations of similar effects in other phage suggest that this may be a general property of phage host range expansion [38], [66]. Similar patterns have been observed following virus adaptation to different cell culture types [67], [68], plant species [54], [55], and animal species [69]. For example, in a host switch of parvoviruses from cats to dogs, the virus responsible for the initial outbreak in dogs (CPV-2) lost the ability to infect cats, although this was later regained [40].

Once a pathogen has infected a new host, the long-term success of the host shift can be independent of reduced performance in the original host if the pathogen does not require transmission to and from the original host for survival. However, trade-offs between performance in the two hosts can prevent adaptation to a new host if the pathogen is transmitted back to the original host at a high rate. For example, a vector borne pathogen may be unlikely to shift from a common mosquito species to a rare one as it will normally end up back in the original mosquito vector. In contrast, directly transmitted pathogens like influenza may be able to establish a continuous transmission chain in the new host, so reduced performance on the original host is not important.

Another important trade-off in the novel host may be between virulence (the harm a pathogen does to a host) and the transmission potential of the pathogen. A number of theoretical and empirical studies have demonstrated that an intermediate level of virulence is often optimal to maximise transmission [70]–[74]. However, following a host shift, a pathogen may produce maladaptive levels of virulence as the novel host–parasite association has not been under direct selection. For example, when the myxoma virus from South American *Sylvilagus* rabbits was transferred to European rabbits (*Oryctolagus cuniculus*), initial case mortality rates were as high as 99.8% in Australia. The rapid mortality is thought to have reduced the window of time that rabbits were able to transmit the virus, and as a consequence virulence rapidly dropped to case mortality rates of ∼90% due to the spread of attenuated virus strains [75], [76]. There appear to be large mutational targets to evolve changes in virulence in the myxoma virus (a DNA virus with a large 162 kb genome), with no mutations common to specific virulence grades [77]. Failure to evolve lower levels of virulence may explain the stuttering chains of transmission seen in some spillover events [78], [79]; however, it is difficult to disentangle whether the low rates of transmission are due to maladaptive levels of virulence or from human intervention.

## Perspectives

One aim of studying host shifts is to predict future disease emergence, but it is unclear whether this will ever be possible with an accuracy that makes it practically useful [80]. While we have rules of thumb as to which groups of pathogens are most likely to host shift, and which donor species they are likely to come from [32], there will always be exceptions. This means that predicting disease emergence by fine scale surveillance of potential donor species and the individuals they are most likely to infect is a hugely difficult task [81]. The observation that specific mutations are often required in host shifts has led to studies looking at whether these mutations can be predicted in advance. For H5N1 avian influenza viruses, mammalian transmissible forms have been evolved in laboratory settings, and identified mutations may be markers for potential epidemics [82]–[84]. However, even in this exceptionally well-studied case, predictive power remains low and highly system specific.

While we increasingly understand the genetic details that underlie host shifts, there are still important questions unanswered. The literature is overwhelmingly skewed towards viruses, but do bacterial and eukaryotic parasites have similar properties? Under what conditions do trade-offs between performance on the original and new host prevent host shifts from happening? Do the mutations involved in host shifts originate as de novo mutations in the new host or come from standing variation in the original host? What determines the size of the mutational target, and does this depend on what the barrier to a host shift is? For example, mutational targets seem to be small for relatively simple traits like changes in receptor use, but may be larger for complex traits like virulence and transmissibility.

One little-explored consequence of host shifts is how they affect the distribution of pathogens across host species [85]. The number and type of pathogens infecting a host is partly a result of past “acquisitions” following host shifts (Box 2), and new theory is showing how our understanding of host shifts can allow us to understand the composition of pathogen communities [24], [86], but these ideas remain largely untested.

**Box 2. Host Shifts Shape the Distribution of Pathogens across Species**

**Figure 3:**
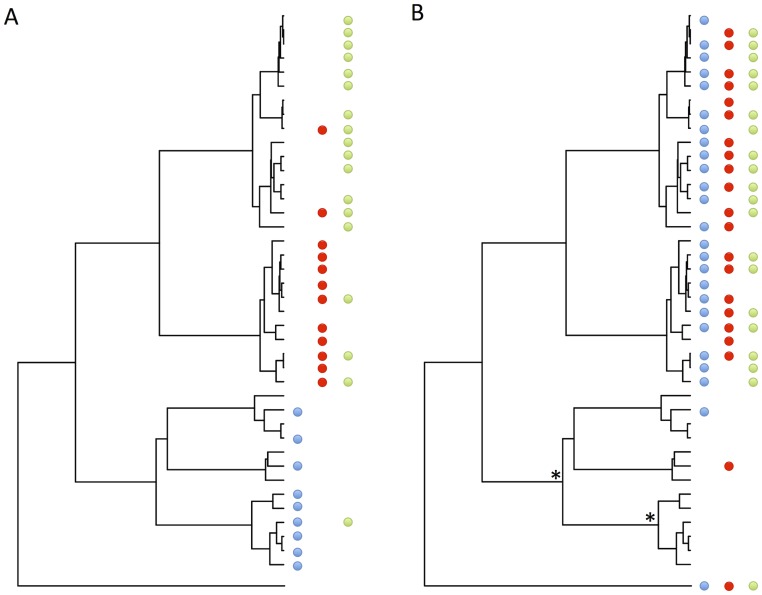
Examples of how patterns of host shifts can affect the distribution of pathogens across the host phylogeny. Each column shows the presence of a different pathogen, with a coloured circle representing the presence of that pathogen. In panel A, pathogens preferentially shift between closely related hosts, while in B closely related host species have similar levels of susceptibility to infection, regardless of the source of the pathogen (with two increases in host resistance occurring at the asterisks on the host phylogeny). Both processes result in closely related host species harbouring similar pathogens, and in some host clades harbouring more pathogen species. However, in A, but not B, host species with more close relatives tend to have more pathogens. For example, the phylogenetically isolated species at the bottom of the tree is not infected by any of the three pathogens in A, but is in B.

The number and taxonomic diversity of pathogens infecting each host species is different, and the processes that shape the composition of these pathogen communities are poorly understood. The distribution of pathogens across host species is the consequence of a balance between the rate at which new host–pathogen associations are “born” following host shifts (or speciation) and “die” when the pathogen goes extinct in a host [24], [85], [86]. If host shifts occur far more frequently than host speciation, then the process is similar to the theory of island biogeography, where species richness of island communities is a balance between the rate at which new species colonise the island and existing species go extinct [88]. Therefore, this hypothesis predicts that species that have properties that increase the rate that pathogens host shift will tend to harbour more pathogens. Because host shifts tend to occur most readily between related species, on the rare occasions a host acquires a pathogen from a distant relative (Figure 1), that pathogen may then rapidly jump into other closely related hosts. This process can generate a clumped distribution of pathogens, where related hosts share related pathogens (Figure 3A) [24], [85]. Furthermore, phylogenetically isolated hosts that have few close relatives are predicted to have fewer pathogens, and their pathogen communities may be dominated by classes of pathogens such as RNA viruses that are prone to move between distantly related hosts. Alternatively, the host phylogeny may consist of clades of intrinsically resistant and susceptible species, and the resistant clades will tend to have lower pathogen diversity (Figure 3B). These processes have been little studied for pathogens [24], but support comes from plant feeding insects, which have lower species richness on phylogenetically isolated hosts [89]. This conceptual framework combined with our understanding of host shifts may prove to be a powerful way to explain the community composition of pathogens.

Finding tractable methods to monitor emerging diseases presents a significant challenge for the future. Studying the genetics of host shifts has the potential to uncover the evolutionary processes that pathogens undergo when they find themselves in a novel host, and may allow us to begin to address this challenge.
